# Validation and characterization of a QTL for adult plant resistance to stripe rust on wheat chromosome arm 6BS (*Yr78*)

**DOI:** 10.1007/s00122-017-2946-9

**Published:** 2017-07-19

**Authors:** Zhenzhen Dong, Joshua M. Hegarty, Junli Zhang, Wenjun Zhang, Shiaoman Chao, Xianming Chen, Yonghong Zhou, Jorge Dubcovsky

**Affiliations:** 1 Department of Plant Sciences, University of California, Davis, CA 95616 USA; 2 Triticeae Research Institute, Sichuan Agricultural University, Wenjiang, Chengdu, 611130 Sichuan People’s Republic of China; 3 USDA-ARS, 1605 Albrecht Blvd, Fargo, ND 58102 USA; 4 Department of Plant Pathology, Washington State University, Pullman, WA 99164 USA; 5 USDA-ARS, Wheat Health, Genetics, and Quality Research Unit, Pullman, WA 99164 USA; 6 Howard Hughes Medical Institute, Chevy Chase, MD 20815 USA

## Abstract

*****Key message***:**

**This study validated one QTL for adult plant resistance to stripe rust, identified donor lines of the resistance allele, and demonstrated that it is different from previously named** ***Yr*** **genes.**

**Abstract:**

The spread of more virulent and aggressive races of *Puccinia striiformis* f. sp. *tritici* (*Pst*, causal pathogen of stripe rust) after the year 2000 has caused substantial yield losses worldwide. To find new sources of resistance, we previously performed a genome-wide association study and identified a strong QTL for adult plant resistance on the short arm of chromosome 6B (*QYr.ucw*-*6B*). In this study, we validated *QYr.ucw*-*6B* in ten biparental populations, and mapped it 0.6 cM proximal to *IWA7257* and 3.9 cM distal to *IWA4408*. We showed that *QYr.ucw*-*6B* is located approximately 15 cM proximal to the all-stage resistance gene *Yr35* and that none of the resistant lines carries the previously cloned *Yr36* gene. Based on these results, *QYr.ucw*-*6B* was assigned the name *Yr78*. This gene was not effective against *Pst* at the seedling stage, suggesting that it is an adult plant resistance gene. *Yr78* has been effective against *Pst* races present in field experiments performed in the Western USA between 2011 and 2016. Since this gene is predicted to be present at low frequency in wheat germplasm from this region, it can provide a useful tool to diversify the sources of resistance against this devastating pathogen.

**Electronic supplementary material:**

The online version of this article (doi:10.1007/s00122-017-2946-9) contains supplementary material, which is available to authorized users.

## Introduction

Stripe rust, caused by *Puccinia striiformis* Westend. f. sp. *tritici* Erikss. (*Pst*), is a devastating disease of wheat. More virulent and aggressive *Pst* races that began to appear around the year 2000 have spread rapidly over most of the wheat growing regions of the world, causing significant yield losses (Chen 2005; Hovmøller et al. 2008, 2016; Milus et al. 2008; Wellings 2011). These new *Pst* races are more aggressive, have broader virulence profiles, and are tolerant to higher temperatures than previously identified races (Markell and Milus 2008; Milus et al. 2008). A comparison with reference isolates sampled from six continents suggested that the post-2011 European races of *Pst* originated from the center of diversity of the pathogen in the near-Himalayan region of South Asia (Ali et al. 2014; Hovmøller et al. 2016).

While stripe rust can be controlled by the timely application of fungicides, control through the deployment of resistant cultivars is a more economically viable and environmentally friendly solution. Unfortunately, many of the previously characterized *Pst* resistance genes are not effective against these new *Pst* races, forcing wheat breeders to look for new sources of resistance. *Pst* all-stage resistance genes *Yr5* (Chen et al. 2003) and *Yr15* (Yaniv et al. 2014) have remained effective to these new races and have been combined into several wheat varieties (e.g. Patwin 515, Summit 515, Blanca Grande 515, etc.). However, the recent release of commercial varieties carrying each of these genes separately is jeopardizing previous gene stewardship strategies and making more urgent the identification of additional resistance genes.

Stripe rust resistance genes are broadly classified into all-stage and adult plant resistance (APR) genes. All-stage resistance genes, which are effective since early stages of plant development, are typically race-specific, and frequently encode NBS-LRR resistance proteins (Lowe et al. 2011). By contrast, APR genes are effective later in development and often, are effective to a broader range of races, confer partial resistance, and encode a more diverse set of proteins (Chen 2013; Fu et al. 2009; Krattinger et al. 2009; Lowe et al. 2011). To expand the list of APR genes against *Pst* in hexaploid wheat (*Triticum aestivum* L.), we previously performed a global genome-wide association study (GWAS) in six different environments in the western USA between the years 2011 and 2013 (Maccaferri et al. 2015).

In this GWAS, we identified ten loci that were highly significant in most of the environments and had a low experiment-wise error rate (Maccaferri et al. 2015). Among these loci, we identified one on chromosome 6B (*QYr.ucw*-*6B*) linked to SNP locus *IWA7257* that was mapped in a different location from previously named *Pst* resistance genes. This locus was highly significant for APR in field trials (infection type *P* < 0.00001), but was not significant at the seedling stage under controlled environments for any of the races (PSTv-4, PSTv-14, PSTv-37, and PSTv-40) tested in the previous study (Maccaferri et al. 2015). This locus was also highly significant in a second GWAS performed in the same study in which accessions with highly resistant infection types (ITs of 0–3) were excluded from the analyses. The last result suggested that this locus confers partial resistance to *Pst* (Maccaferri et al. 2015).

Even though the previous GWAS provided valuable information about the chromosome locations of multiple QTL, an experimental validation is still required to identify which of the accessions carrying the favorable SNP allele actually carry the associated resistance gene. This validation is particularly important for wheat breeders to identify germplasm sources from which the resistance alleles can be introgressed into their breeding programs. The identification of correct donor lines is also critical to develop high-density genetic maps and, eventually, to clone the resistance gene. GWAS in wheat are usually insufficient to identify candidate genes because linkage disequilibrium in this species frequently extends over several cM due to its self-pollinating nature and recent origin (Chao et al. 2010; Maccaferri et al. 2015).

In this study, we report the validation of *QYr.ucw*-*6B* in multiple biparental populations and the identification of multiple donors of the resistance alleles that can be used as parental lines in wheat breeding programs. We also report the difference between this QTL and previously named *Pst* resistance genes on chromosome arm 6BS, which supports its official designation as *Yr78* (*QYr.ucw*-*6B*). We show that this gene is more effective in adult plants, and propose that it will constitute a useful tool for wheat breeding programs interested in diversifying the sources of genetic resistance against the new virulent races of *Pst*.

## Materials and methods

### Development of biparental populations

From the GWAS described above (Maccaferri et al. 2015), we selected 10 accessions that carry the resistance allele for *QYr.ucw*-*6B* (Table 1). All of these accessions are publicly available in the United States Department of Agriculture (USDA) National Small Grains Collection (NSGC). Table 1 summarizes their accession numbers, country of origin, year received by the National Plant Germplasm System and alleles present in the markers at the peak of the QTL.

**Table 1:** Accessions used as resistant parental lines in the validation populations listed with their geographical origins, the year received by the National Plant Germplasm System, and allele for the SNP marker at the peak of the QTL

ID	Country	Year	Evaluation	Allele	Infection type (IT)		Severity (SEV)	
*QYr.ucw*-*6B* (Avocet ‘S’ *IWA7257* = GG)				*IWA7257*	*R* ^2^ (%)	*P*	*R* ^2^ (%)	*P*
PI 192493	Mozambique	1950	2016	TT	13.0	0.0022	12.7	0.0025
PI 519805	Uruguay	1987	2015/2016	TT	51.3	<0.0001^1^	48.9	<0.0001
PI 494101	U.S.A.	1984	2015/2016	TT	16.5	0.0003	14.6	0.0009
PI 532116	Egypt	1988	2015/2016	TT	14.6	0.0026^1^	11.0	0.0055^1^
PI 191351	Russia	1950	2015/2016	TT	10.1	0.0190^1^	6.8	0.038^1^
PI 286543	Ecuador	1963	2015/2016	TT	19.3	<0.0001	18.9	<0.0001
PI 351878	Burundi	1969	2015/2016	TT	21.9	0.0001^1^	22.0	0.0002^1^
PI 520108	Mexico	1987	2015/2016	TT	11.4	0.0209^1^	10.2	0.0323^1^
PI 520265	U.S.A.	1988	2016	TT	29.0	<0.0001	24.0	<0.0001
PI 520378	Syria	1988	2016	TT	27.3	<0.0001	29.6	<0.0001

Each accession was crossed with susceptible variety Avocet ‘S’ and one-way ANOVAs were performed for adult plant infection type (IT) and severity (SEV) in the field for the marker at the peak of the QTL. Percent variation explained by the peak marker (*R* ^*2*^) and the associated probabilities (*P* values) are presented in the last columns

^1^ These *P* values were calculated using non-parametric Kruskal–Wallis tests due to lack of normality. *R* ^2^ values were obtained from the parametric ANOVAs

The putative donors of the resistance allele were crossed with the susceptible variety Avocet ‘S’, which has been used as a recurrent parent in the generation of near isogenic lines for many *Yr* genes (McIntosh et al. 1995; Wan and Chen 2014). The F_1_s were self-pollinated in the greenhouse and F_2_ populations were developed, including 94 individuals each. The first seven F_2_ populations were evaluated during the 2014–2015 growing season and F_3_ families derived from F_2_ plants homozygous for the peak marker were evaluated during the following growing season. Three additional F_2_ populations for *QYr.ucw*-*6B* were evaluated only in the 2015–2016 growing season. Plants were genotyped for *IWA7257*, the marker associated with the peak of *QYr.ucw*-*6B*.

All field trials were performed at the University of California Experimental Field Station in Davis, California (38°31′33″N, 121°46′30″W, elevation 16 m; henceforth UCD). The experiments were surrounded with a susceptible border (D6301) that was inoculated with *Pst* spores collected from the previous field season.

### Genotyping of the biparental populations

Leaf tissues from individual F_2_ plants and their respective parental lines were collected at the five-leaf stage. Genomic DNA was extracted using previously published methods (Anderson et al. 1993). All the bi-parental populations were genotyped using a KASP assay (Kompetitive Allele Specific PCR, LGC-Genomics, UK) (Semagn et al. 2014). The targeted SNP was at the 3′ end of the primers, following standard KASP guidelines. The allele-specific primers for *IWA7257* (*QYr.ucw*-*6B*) were IWA7257_Rev_A_VIC (GAAGGTCGGAGTCAACGGATTagaccctacgacgttagcga) and IWA7257_Rev_C_FAM (GAAGGTGACCAAGTTCATGCTagaccctacgacgttagcgc), with common primer IWA7257_Com1 (attggaatcagctgggtcat). The capital letters of the primers indicate the VIC and FAM tails and the 3′ allele-specific nucleotide is underlined. The length of the amplicon was 77 bp.

The primer assay mix (100 µl) included 12 µl VIC primer (100 mM), 12 µl FAM primer (100 mM), 30 µl common primer (100 mM), and 46 µl distilled water. KASP assays were performed in a 5.07 µl reaction volume (2.5 µl 2× KASP Master Mix, 0.07 µl KASP primer assay mix and 2.5 µl genomic DNA at 5–50 ng µl^−1^). A two-step touchdown PCR was carried out using the following conditions: 94 °C for 15 min, followed by ten cycles of touchdown of 94 °C for 20 s, annealing from 61 to 55 °C for 1 min (dropping 0.6 °C per cycle), followed by 26 cycles of 94 °C for 20 s, annealing at 55 °C for 1 min. KASP results were analyzed with a FLUOstar Omega F plate reader (BMG LABTECH, Ortenberg, Germany) using the software KlusterCaller (LGC Genomics, Teddington, UK).

For the PI 519805 × Avocet ‘S’ population we developed a complete genetic map to identify plants carrying only the resistance gene underlying *QYr.ucw*-*6B*. This population was genotyped using the Infinium wheat SNP 9 K iSelect assay (Illumina Inc., San Diego, CA, USA) (Cavanagh et al. 2013) at the USDA-ARS genotyping laboratory at Fargo, North Dakota. A total of 2821 polymorphic SNP markers were obtained and used for genetic map construction. The map of the 6B chromosome was supplemented with six polymorphic simple sequence repeats (SSRs, GrainGenes database http://wheat.pw.usda.gov/GG3/).

### Field inoculation and evaluation of stripe rust severity

All populations were sown in the field in mid-November surrounded by *Pst* susceptible borders and interspersed with susceptible plants to facilitate the spread of the disease. Susceptible spreader lines were also planted between every population. The F_2_ populations were evaluated as single plants in 1-m rows including five plants per row, with a separation of 30 cm between rows to facilitate disease evaluation. Individual F_2_ plants were genotyped with *IWA7257*. Selected F_3_ families derived from F_2_ lines homozygous for *IWA7257* were evaluated in rows with at least 30 plants each in the 2015–2016 growing season.

The field was inoculated in February with a mixture of *Pst* spores from the previous year. The *Pst* races collected from infected wheat plants at the UC Davis field in 2015 and 2016, and their virulence formulas are described in Table 2. During both growing seasons, all susceptible spreader rows and borders became fully infected with *Pst*, providing a strong and uniform disease pressure.

**Table 2:** Virulence formulas (Wan and Chen 2014) for *Pst* races detected in the UC Davis field during the 2014–2015 and 2015–2016 growing seasons

PSTv race	Year collected	Virulence formula
PSTv-11	2016	*Yr1, Yr6, Yr7, Yr8, Yr9, Yr17, Yr28, Yr43, Yr44, YrExp2, YrTye*
PSTv-15	2016	*Yr1, Yr6, Yr7, Yr9, Yr17, Yr27, Yr43, Yr44, YrSP, YrExp2, YrTye*
PSTv-17	2016	*Yr1, Yr6, Yr7, Yr8, Yr9, Yr17, Yr27, Yr43, Yr44, YrSP, YrExp2, YrTye*
PSTv-30	2015	*Yr6, Yr7, Yr8, Yr9, Yr44, YrTr1, YrExp2*
PSTv-37	2015 and 2016	*Yr6, Yr7, Yr8, Yr9, Yr17, Yr27, Yr43, Yr44, YrTr1, YrExp2*
PSTv-52	2015	*Yr6, Yr7, Yr8, Yr9, Yr17, Yr27, Yr43, Yr44, YrExp2*
PSTv-53	2015 and 2016	*Yr1, Yr6, Yr9, YrSP, YrTye*
PSTv-142	2015	*Yr6, Yr7, Yr9, Yr44, YrTye*

Wheat lines were evaluated for *Pst* resistance twice, approximately two weeks before and two weeks after heading, to minimize potential escapes, and to avoid the death of plants severely affected by the disease before the scoring. The second scoring date occurred during heavier infection and was preferred for the statistical analysis. Infection type (IT) was scored using the McNeal’s 0 (resistant)–9 (susceptible) scale (Line and Qayoum 1992). Disease severity (SEV) was scored as the percentage of leaf area infected.

### Evaluation of seedling resistance to *Pst*

To test the effect of *QYr.ucw*-*6B* on seedling resistance, we selected four BC_1_F_2_ plants derived from the cross PI 519805/2*Avocet ‘S’ that were homozygous for the *QYr.ucw*-*6B* resistance allele, and homozygous for the susceptible allele for the other resistance QTL detected in this population, and inoculated them with *Pst* spores collected in the field in 2015. As positive control, we used a line homozygous for *Yr35* that was generously provided by Professor Robert Park (Plant Breeding Institute, University of Sydney). In addition, we evaluated seedlings from two F_3_ families homozygous for the *QYr.ucw*-*6B* resistance allele, three homozygous for the susceptibility allele, one line homozygous for the all-stage resistance gene *Yr35*, and the susceptible control Avocet ‘S’ with races PSTv-4, PSTv-14, PSTv-37, PSTv-40 and PSTv-51 at Washington State University.

*Pst* reactions at the seedling stage were evaluated in a CONVIRON growth chambers (PGR15) using protocols similar to those described before (Chen et al. 2002; Pahalawatta and Chen 2005). Briefly, plants were inoculated at the two-leaf stage using spores collected in the field in 2015, and were kept in the dark at 90% humidity and 10 °C for 48 h. Plants were then transferred into a diurnal temperature cycle that changed gradually from 10 to 20 °C with 16 h photoperiod. The chambers had metal halide and high-pressure sodium light that provided a light intensity of ~260 µM m^−2^ s^−1^. Infection types (ITs) were recorded 20–22 days after inoculation using the McNeal’s 0–9 scale (Line and Qayoum 1992).

### Statistical and QTL analyses

For each population segregating for *QYr.ucw*-*6B*, the genotype at the marker *IWA7257* was used as a class variable in one-way ANOVAs for infection type and severity. Normality of residuals was tested using the Shapiro–Wilk test and homogeneity of variances using Levene’s tests as implemented in SAS 9.4 (SAS Institute Inc., Cary, NC, USA). For those populations that did not meet these assumptions, we first tested different power transformations, and if they still did not meet the assumptions, we analyzed those populations using a non-parametric test (Kruskal–Wallis, as implemented in SAS 9.4).

For the population PI519805 × Avocet ‘S’ segregating for *IWA7257*, a genetic map was constructed with JoinMap 4.0 (Van Ooijen 2006) and MAPMAKER/EXP 3.0 (Lincoln et al. 1992) using the Kosambi mapping function (Kosambi 1944). A minimum LOD (logarithm of odds) threshold of 2.0 was used for grouping markers into linkage groups, and a three-point linkage analysis was carried out to determine the most likely order of linked markers. Linkage groups were assigned to chromosomes using a previous consensus map (Cavanagh et al. 2013). Completely linked markers were merged and only one of the markers for each group was used for the QTL analysis. For groups of linked markers known to be in the same chromosome but for which no significant linkage was detected between groups, the distances were estimated from the consensus map (Cavanagh et al. 2013). Those estimated distances are indicated in parenthesis and marked with an “*” in the maps.

QTL analyses were performed with Windows QTL Cartographer V2.5 (Wang et al. 2012) using composite interval mapping (CIM) with both backward and forward regressions at *α* = 0.1. A threshold (LOD) value of 3.0 was used to determine the presence of significant QTL.

## Results

### Validation populations for *QYr.ucw*-*6B* (*Yr78*)

The ten accessions selected for *Yr78* showed good adult plant resistance in the field (supplementary Fig. S1 A–J). In all ten segregating populations derived from these accessions, we detected significant effects associated with SNP marker *IWA7257* (peak marker of *QYr.ucw*-*6B* in GWAS) for both *Pst* infection type and severity (Table 1). This result suggests that *IWA7257* is a relatively good predictor of *QYr.ucw*-*6B*. The association between *IWA7257* and *Pst* resistance was further validated during the 2015–2016 growing season using F_3_ families derived from homozygous F_2_ plants. All seven F_3_ populations showed highly significant differences in *Pst* infection type and severity between families homozygous for the different *IWA7257* alleles (Table 3).

**Table 3:** One-way analysis of variance for adult plant infection type (IT) and severity (SEV) for *IWA7257* (linked to *Yr78*) in seven F_3_ populations

*QYr.ucw*-*6B*	*N*	IT *R* ^2^ (%)	IT *P*	SEV *R* ^2^ (%)	SEV *P*
PI 191351	42	48.7	<0.0001	39.6	<0.0001
PI 286543	56	60.5	<0.0001	54.3	<0.0001
PI 351878	34	50.0	<0.0001	47.1	<0.0001
PI 520108	34	25.6	0.0022	33.1	0.0004
PI 519805	41	74.4	<0.0001	73.6	<0.0001
PI 494101	37	31.4	0.0003	26.4	0.0012
PI 532116	46	41.1	<0.0001	37.4	<0.0001
Average		47.4		44.5	

Accessions listed in the first column are the female parents of the crosses with susceptible variety Avocet ‘S’. Percentage of variation explained by the peak marker (*R* ^2^) and the associated probabilities (*P* values) recorded in adult plants in the field at UC Davis are presented in the last columns

On average, the *IWA7257* marker explained 21.4 ± 3.9% of the variation in IT and 19.9 ± 3.9% of the variation in severity in the F_2_ experiment (Table 1) and 47.4 ± 6.3% of the variation in IT and 44.5% ± 5.9% of the variation in severity in the F_3_ experiment (Table 3). In both the F_2_ and F_3_ experiments, the population generated from the cross PI 519805 × Avocet ‘S’ showed the largest proportion of variation explained by the *IWA7257* marker (Tables 1, 3), and was selected for the construction of a complete linkage map, and for the identification of lines segregating only for *QYr.ucw*-*6B*.

### PI 519805 × Avocet ‘S’ linkage map

We generated a complete linkage map of the PI 519805 × Avocet ‘S’ population to evaluate the presence of other *Pst* resistance QTL. This information was used to select plants segregating only for *QYr.ucw*-*6B* for further evaluation with different *Pst* races. We genotyped the complete F_2_ population using the Illumina iSelect 9K SNP assay (Cavanagh et al. 2013), six SSR markers from chromosome 6B, two from chromosome 6D (Somers et al. 2004), and marker *csLV46* (Dr. Evans S Lagudah, personal communication). Marker csLV46 was previously mapped linked to *Yr29* on chromosome arm 1BL (Kolmer et al. 2015; Lan et al. 2014). A total of 2821 polymorphic SNP markers were identified, and mapped to 689 unique loci covering 2425 cM. Linkage groups were assigned to chromosomes based on previously published consensus SNP maps (Cavanagh et al. 2013). Fewer SNP markers were mapped on chromosomes from the D genome (221 markers) than on those from the A (1072 markers) or B (1491 markers) genomes (37 markers were ungrouped). A lower proportion of markers in the D genome was also reported for the SNP consensus map, which was generated with the same SNP assay (Cavanagh et al. 2013).

Markers from chromosomes 5B and 7B were linked in this population suggesting that PI 519805 (ND 457*3/*T.durum*//Estanzuela Dakuro) carries the centromeric reciprocal translocation 5BL-7BL and 5BS-7BS, which was first identified in *T. aestivum* ssp. *compactum* (Sears 1953) and was prevalent in West European wheats in the 1960s and 1970s and in their descendants (Riley et al. 1967). The short and long arms of the 5B and 7B maps were manually separated and presented without the translocation in supplementary Fig. S2.

**Fig. 1:**
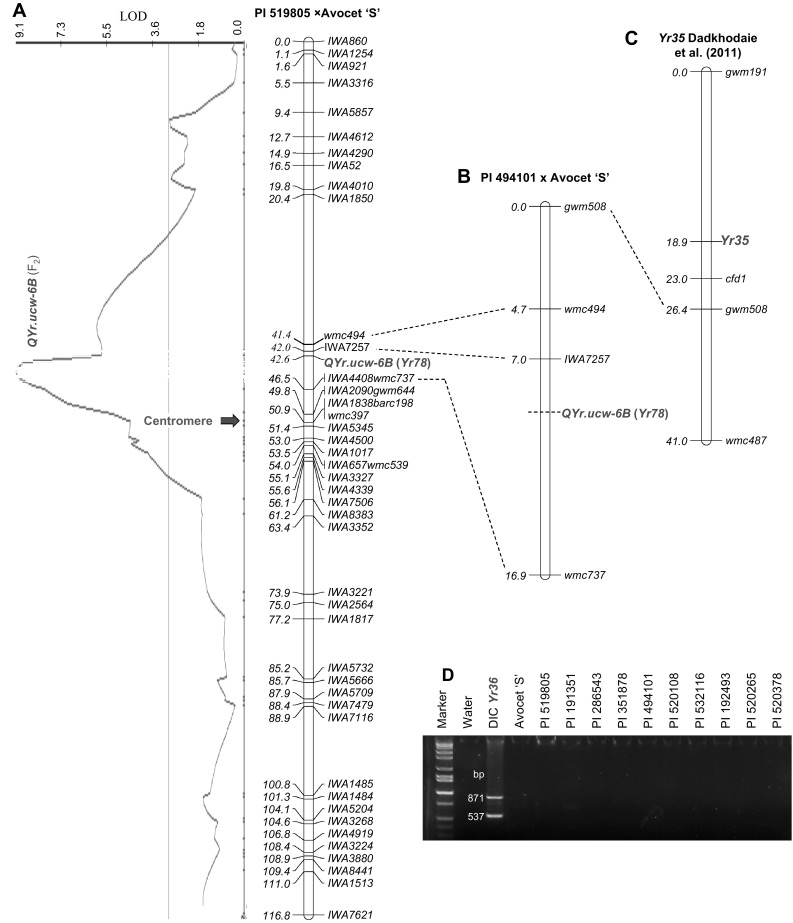
Comparative maps of *QYr.ucw*-*6B* and *Yr35.* **a** Linkage map and QTL analysis based on F_2_ plants from the population PI 519805 × Avocet ‘S’. **b** Linkage map of the population PI 494101 × Avocet ‘S’. The position of *QYr.ucw*-*6B* as a Mendelian gene in the two linkage maps is based on F_3_ field data. **c** Map of *Pst* all-stage resistance gene *Yr35* based on Dadkhodaie et al. (2011) including common marker *gwm508*. **d** Evaluation of the presence of *Yr36* in the ten lines confirmed to carry *QYr.ucw*-*6B*. DIC = *T. turgidum* ssp. *dicoccoides* carrying the *Yr36* gene (positive control)

In this population, *IWA7257* was mapped on the short arm of chromosome 6B, 0.6 cM proximal to SSR marker *wmc494* and 4.5 cM distal to linked markers *IWA4408* (SNP) and *wmc737* (SSR) (Fig. 1a). This differs from the position of *IWA7257* on the long arm of chromosome 6B (scaled position 112.3 cM) in the 9K iSelect assay SNP consensus map (Cavanagh et al. 2013). A comparison of the 200 bp sequence flanking SNP *IWA7257* with the IWGSC draft sequence of the different chromosome arms of Chinese Spring (https://urgi.versailles.inra.fr/blast/blast.php) showed 100% identity to sequences from chromosome arm 6BS (BLASTN *E* = 3e^−98^) and no significant similarity to 6BL, which confirmed that *IWA7257* is located on the short arm of chromosome 6B.

### PI 519805 × Avocet ‘S’ QTL analysis

Using a threshold LOD score of 3.0, we identified two significant QTL for infection type and severity on chromosome arms 6BS (*QYr.ucw*-*6B*, Fig. 1a) and 1BL (*QYr.ucw*-*1B*, supplementary Fig. S3). In a factorial ANOVA including both QTL, *QYr.ucw*-*6B* explained 45.9% of the variation in IT and 49.7% of the variation in severity (slightly different from the values in Table 1 calculated from one-way ANOVAs), whereas *QYr.ucw*-*1B* explained 30% of the variation in IT and 27% of the variation in severity. No significant interaction was detected between these two QTL suggesting that their contributions to *Pst* resistance are mainly additive (independent from each other).

The peak of *QYr.ucw*-*6B* (based on F_2_ data), was mapped between markers *IWA7257* and *wmc737/IWA4408* on chromosome arm 6BS (Fig. 1a). This location was confirmed using data from F_3_ families homozygous for the resistance and susceptibility alleles. In population PI 519805 × Avocet ‘S’, *QYr.ucw*-*6B* was mapped 0.6 cM proximal to *IWA7257* and 3.9 cM distal to *wmc737/IWA4408* (Fig. 1a). Although a similar location was detected for population PI 494101 × Avocet ‘S’ (Fig. 1b), the position of the QTL in the PI 519805 × Avocet ‘S’ map is probably more precise because we had information of the additional resistance allele segregating in this population. In summary, the results of the three mapping experiments consistently indicate that *QYr.ucw*-*6B* is located within the *IWA7257*-*wmc737* interval. We analyzed each of the critical recombination events and provided our best estimate of the position of the underlying *Yr78* gene within this interval (Fig. 1a, b). However, the results presented here should be considered only as a first step in the Mendelization of *QYr.ucw*-*6B*, because these populations were not generated from isogenic lines, and segregation for other *Pst* resistance genes in the background can generate some imprecisions in our mapping results.

The QTL mapped on the distal region of chromosome arm 1BL (peak at SNP marker *IWA802*, LOD = 5.7) was designated as *QYr.ucw*-*1B* based on its similar position to a QTL mapped in a previous GWAS (Maccaferri et al. 2015). *IWA802* was mapped 3 cM proximal to *csLV46* (supplementary Fig. S3), a marker that has been previously mapped linked to the leaf rust/stripe rust APR locus *Lr46/Yr29* (Kolmer et al. 2015). Based on the comparison with the map published by Kolmer et al. (2015), it is likely that *QYr.ucw*-*1B* corresponds to *Yr29* (supplementary Fig. S3).

### Comparison of *QYr.ucw*-*6B* with other *Pst* resistance genes on chromosome arm 6BS

To determine if *QYr.ucw‐6B* was different from *Pst* resistance genes *Yr35* and *Yr36* previously mapped on the same chromosome arm, we compared their positions on the 6BS map and their response to different *Pst* races at the seedling stage.

The all-stage resistance gene *Yr35* was previously mapped between SSR markers *gwm191* and *cfd1* (Dadkhodaie et al. 2011). Although we were unable to map these two SSR markers in our segregating populations, we were able to map g*wm508* (the next SSR marker proximal to *Yr35*) in the population PI 494101 × Avocet ‘S’ (Fig. 1b). *Yr35* was mapped 7.5 cM distal to *gwm508,* whereas *IWA7257* was mapped 7 cM proximal to *gwm508* (Fig. 1c). Since *QYr.ucw‐6B* was mapped proximal to *IWA7257*, the previous results indicate that *QYr.ucw‐6B* is at least 15 cM proximal to *Yr35* (compare Fig. 1b and c), and that they are different genes. This hypothesis is also supported by different reactions to *Pst* inoculations at the seedling stage described in the next section.

The *Yr36* gene was previously cloned and was shown to encode a protein carrying a START and a kinase domain, designated WKS1 (Fu et al. 2009). We used primers specific for the *WKS1* START domain (Fu et al. 2009) to test for the presence of this gene in the ten populations segregating for *Yr78*. We amplified the expected 871- and 537-bp PCR fragments from the positive control line *T. turgidum* ssp. *dicoccoides* accession FA15-3 (DIC, Fig. 1d). The 871-bp fragment corresponds to *WKS1* and the 537-bp to the closely linked non-functional paralog *WKS2* (Gou et al. 2015). The ten lines carrying the *QYr.ucw*-*6B* resistance allele (Table 1) did not show any PCR amplification product using the same set of primers (Fig. 1d). This result confirmed that *Yr36* is not present in these lines and that, therefore, *QYr.ucw*-*6B* and *Yr36* are different genes. Based on the *Yr35* and *Yr36* results, *QYr.ucw*-*6B* was assigned the official name *Yr78*.

### Seedling reaction for *QYr.ucw*-*6B*

The ten lines validated for the presence of *Yr78* (Table 1) showed relatively high levels of resistance to *Pst* in the field at the adult plant stage (supplementary Fig. S1, A–J). At the seedling stage, all BC_1_F_2_ plants from the cross PI 519805/2*Avocet ‘S’ homozygous for *Yr78* were as susceptible to *Pst* (field collected spores, supplementary Fig. S1 K) as the susceptible control Avocet ‘S’ (supplementary Fig. S1 K). By contrast, seedlings homozygous for *Yr35* (Dadkhodaie et al. 2011) grown in the same chamber showed a clear resistance reaction to the same *Pst* spores (supplementary Fig. S1 K).

Similar results were obtained for F_3_ seedlings from the PI519805 × Avocet ‘S’ cross inoculated with *Pst* races PSTv-4, PSTv-14, PSTv-37, PSTv-40, and PSTv-51 in a growth chamber (Table 4). F_3_ seedlings homozygous for *Yr78* and those homozygous for the susceptibility allele, both showed high levels of infection type (mainly ITs 7 and 8), similar to those observed in the susceptible recurrent parent Avocet ‘S’ (Table 4). As in the experiment using *Pst* urediniospores collected in the field, the seedlings carrying *Yr35* showed high levels of resistance to the five individual races (mainly ITs 1–3). These results confirmed that *Yr35* and *Yr78* are different genes and that *Yr78* is an APR gene.

**Table 4:** Comparison of infection types in seedlings from a line homozygous for *Yr35* and from F_3_ plants (cross PI 519805 × Avocet ‘S’) homozygous for the resistance (R) and susceptibility (S) alleles of SNP marker *IWA7257* (linked to *Yr78*)

	PSTv-4	PSTv-14	PSTv-37	PSTv-40	PSTv-51
*Yr35* (homozygous)	2(14),3(5)	2(11),3(6),5(3)	2(9),3(10)	1(3),2(17),3(2)	2(17),3(2)
*IWA7257* TT (R)	8(21)	8(19)	8(21)	5(2),7(5),8(12)	7(5),8(15)
*IWA7257* TT (R)	8(19)	8(21)	8(20)	7(4),8(14)	7(5),8(15)
*IWA7257* GG (S)	8(21)	8(21)	8(20)	8(19)	8(22)
*IWA7257* GG (S)	8(20)	8(21)	8(21)	8(20)	7(3),8(16)
*IWA7257* GG (S)	8(21)	8(20)	8(21)	7(5),8(15)	8(20)
Avocet ‘S’	8(20)	8(20)	8(20)	8(20)	8(20)

All the selected F_3_ plants were homozygous for the susceptibility allele of *QYr.ucw*-*1BL* located on the long arm of chromosome 1B

Infection types (ITs) recorded 20–22 days after inoculation using McNeal’s 0–9 scale (Line and Qayoum 1992). Numbers in parentheses indicate the number of tested plants

## Discussion

New *Pst* races that appeared around the year 2000 in the USA and later in Australia, Europe and North Africa have a broader virulence profile, increased aggressiveness and better tolerance to high temperatures than previous races. These races have defeated many of the previously known stripe rust resistance genes, depleting the options available to wheat breeders. To expand the set of resistance genes we performed a GWAS and identified several potential new genes (Maccaferri et al. 2015). In this study, we validated and characterized one of these resistance genes on wheat chromosome arm 6BS (*Yr78*), and showed that it is different from stripe rust resistance genes previously identified in this chromosome arm.

### Relation between *Yr78* and other resistance genes and QTL mapped to chromosome arm 6BS

New official *Yr* names are assigned to *Pst* resistance loci that are different from previously named *Yr* genes, and for which donor lines are available in public seed repositories. *QYr.ucw‐6B* satisfied both requirements. The donor lines of this *Pst* resistance QTL are all deposited in the USDA National Small Grains Collection, and are available upon request. In addition, we showed that *Yr78* is different from *Pst* resistance genes *Yr35* and *Yr36*, previously mapped on chromosome arm 6BS.

Using a common SSR marker, we mapped *Yr78* roughly 15 cM proximal to the known location of *Yr35* (Fig. 1), and showed that, at the seedling stage, these two genes differ in their reactions to multiple *Pst* races. Seedlings carrying *Yr35* were resistant to all tested *Pst* races, whereas seedlings carrying *Yr78* showed infection types and severities similar to those observed in the susceptible parent Avocet ‘S’ (supplementary Fig. S1). Finally, *Yr35* was transferred to hexaploid wheat from *T. turgidum* ssp. *dicoccoides* (Marais et al. 2005), several years after the development of the cultivars carrying *QYr.ucw*-*6B* reported in this study (Table 1). Taken together, the previous arguments consistently support the hypothesis that *Yr78* and *Yr35* are different genes.

We also demonstrated that *QYr.ucw*-*6B* is different from the previously cloned resistance gene *Yr36* (Fu et al. 2009) since primers diagnostic for *Yr36* failed to amplify any products in the lines validated for *QYr.ucw*-*6B* in this study (Fig. 1d). Based on the previous results *QYr.ucw*-*6B* was officially assigned the name *Yr78*.

We also explored QTL previously mapped close to the *Yr78* region to determine if they are synonymous of the new designated *Yr* gene. Of the five QTL previously mapped close to the region including *Yr78* on chromosome 6B, three were mapped on the long arm, and are likely different genes. These include *QYr.caas*-*6B* (Lan et al. 2010), *QYr*-*6B* (William et al. 2006), and *QYr.inra*-*6B* (Dedryver et al. 2009). By contrast, *QYr.sun*-*6B* identified in the Australian variety Janz (Bariana et al. 2010) and *QYr.wgp*-*6BS.1* identified in the US wheat variety Stephens (Santra et al. 2008) were both mapped on the 6BS arm distal to *gwm644*, within regions that overlap with *Yr78* (Fig. 1a). Janz and Stephens, both carry the “T” allele at *IWA7257* (=AA), which accurately predicted the presence of *Yr78* in the ten populations validated in this study. Based on these results, we propose that both Janz and Stephens carry *Yr78*, and suggest that *QYr.sun*-*6B* and *QYr.wgp*-*6BS.1* be designated as synonymous of *Yr78*. To the best of our knowledge, *QYr.wgp*-*6BS.1* should be credited as the first report for *Yr78* (Santra et al. 2008).

*IWA7257*, the closest marker to *Yr78*, was also reported to be significantly associated with *Pst* resistance in two GWAS conducted in the Pacific North West (PNW). The first one, used a core subset of 1175 accessions from the NSGC winter wheat germplasm collection (Bulli et al. 2016), and the other one used 402 advanced winter wheat accessions from the PNW winter panel (Naruoka et al. 2015). These results suggest that *Yr78* is present in both spring and winter wheat cultivars.

### All-stage versus partial adult plant resistance genes

In the two GWAS for winter wheat (Bulli et al. 2016; Naruoka et al. 2015), as well as in the GWAS for spring wheat (Maccaferri et al. 2015), the *IWA7257* marker was associated with APR to *Pst* in the field, but not with seedling resistance in inoculations performed in controlled environment experiments (Table 4; supplementary Fig. S1). In this study, we confirmed that *Yr78* is not effective at the seedling stage, but that it confers partial resistance to *Pst* at the adult plant stage (supplementary Fig. S1).

The classification of *Yr78* as a partial APR gene has some practical implications. First, the few cloned wheat genes conferring partial resistance to *Pst* at the adult plant stage, have revealed protein architectures different from the canonical NBS-LRR resistance genes (Fu et al. 2009; Krattinger et al. 2009). The identification of such genes provides the opportunity to diversify the resistance mechanisms used to control this rapidly evolving pathogen. In addition, some of these partial APR genes have been historically more durable than many of the all-stage resistance genes, which can be rapidly defeated by changes or deletions of the recognized effectors (Lowe et al. 2011). The classification of *Yr78* as a partial APR gene will likely increase the interest in cloning this gene.

### Introgression of *Yr78* into breeding programs

The “T” (=AA) allele of *IWA7257* was predictive of the presence of the resistance gene in all ten populations selected for validation in this study. This allele was also predictive of the presence of a *Pst* resistance QTL on 6BS (likely *Yr78*) in the varieties Janz (Bariana et al. 2010) and Stephens (Santra et al. 2008). These results suggest that *IWA7257* is genetically close to *Yr78* and that limited historical recombination has occurred between this marker and the resistance gene. Even though the “T” allele of *IWA7257* has been a good predictor of the presence of *Yr78*, we have found some putative recombination events between *IWA7257* and *Yr78* in our segregating populations. This suggests that *IWA7257* is not a perfect marker for *Yr78*, and that it should be used cautiously to predict the presence of *Yr78* in uncharacterized germplasm.

Among the winter wheat varieties, the frequency of the *IWA7257* “T” allele associated with *Pst* resistance was 24% in the NSGC winter wheat core collection (1175 genotyped accessions) (Bulli et al. 2016), and 46% in the PNW winter panel (Naruoka et al. 2015). Among the spring wheats in the NSGC core collections, the *IWA7257* “T” allele was present on average in 23% of the hexaploid wheat accessions and 1.3% of the tetraploid accessions. Therefore, selection for *Yr78* can be used to improve *Pst* resistance in a large number of lines in both tetraploid and hexaploid wheat breeding programs and to diversify the *Pst* resistance genes currently used to control this pathogen.

So far, the effectiveness of *Yr78* against the new *Pst* races has been demonstrated only in the western USA and Australia (Bariana et al. 2010). Therefore, before deploying this gene in other regions, it will be necessary to confirm its effectiveness against the local *Pst* races. If effective, *Yr78* will particularly useful in Asia, where the favorable *IWA7257* allele is present at low frequency (<10%) (Maccaferri et al. 2015).

In summary, *Pst* resistance gene *Yr78* provides wheat breeders with a new tool to control the devastating stripe rust disease. Since *Yr78* is in the same chromosome arm as are *Yr35* and *Yr36* it would be useful to combine *Yr78* with *Yr35* and *Yr36* in phase to facilitate their simultaneous introgression into commercial wheat varieties.

#### **Author contribution statement**

ZD evaluated the populations, analyzed the data and wrote the first draft. JH performed the crosses, developed the mapping populations and contributed to the QTL analysis. JZ contributed to the statistical and QTL analyses. WZ contributed to the construction of the genetic maps. SC provided the SNP genotyping. XC performed the seedling resistance tests and the determination of the *Pst* races present in the field. YZ supervised ZD and revised the manuscript. JD suggested the project, contributed to the genetic map and statistical analyses and generated the final version of the manuscript. All authors revised the manuscript and provided suggestions.

## Electronic supplementary material

Below is the link to the electronic supplementary material.

Supplementary Fig. S1. a-j Adult plant resistance in the field for lines validated for Yr78 (QYr.ucw-6B) compared with susceptible control Avocet ‘S’. “F1″ indicate hybrids between the PI accession carrying Yr78 and Avocet ‘S’. k Seedling resistance test in a growth chamber inoculated with field spores. The first three leaves are from plants carrying the all-stage resistance gene Yr35, the fourth leaf is from the susceptible control Avocet ‘S’, and the four last leaves are from BC1F2 plants from population PI 519805/2*Avocet ‘S’ homozygous for Yr78 (and homozygous for the QYr.ucw-1B susceptibility allele).

Supplementary Fig. S2. Linkage map of population PI 519805 × Avocet ‘S’. The 5B and 7B maps were linked due to a reciprocal translocation and were manually separated. For groups of completely linked SNP markers, only one member of the group is presented for simplicity.

Supplementary Fig. S3. QTL analysis of chromosome 1BL in population PI 519805 × Avocet ‘S’ and its comparison with the QTL map of Yr29 in RILs from the population Tc*3/CI13227 (Kolmer et al. 2015).

Supplementary material 1 (PDF 813 kb)
