# Supplementary material for: Validation and characterization of a QTL for adult plant resistance to stripe rust on wheat chromosome arm 6BS (Yr78)
Source: Theor Appl Genet. 2017 Jul 19;130(10):2127–37. doi: 10.1007/s00122-017-2946-9 (PMC5606939; doi:10.1007/s00122-017-2946-9)
Supplement: Supplementary file 1 — Supplementary material 1 (PDF 813 kb) [file 122_2017_2946_MOESM1_ESM.pdf]

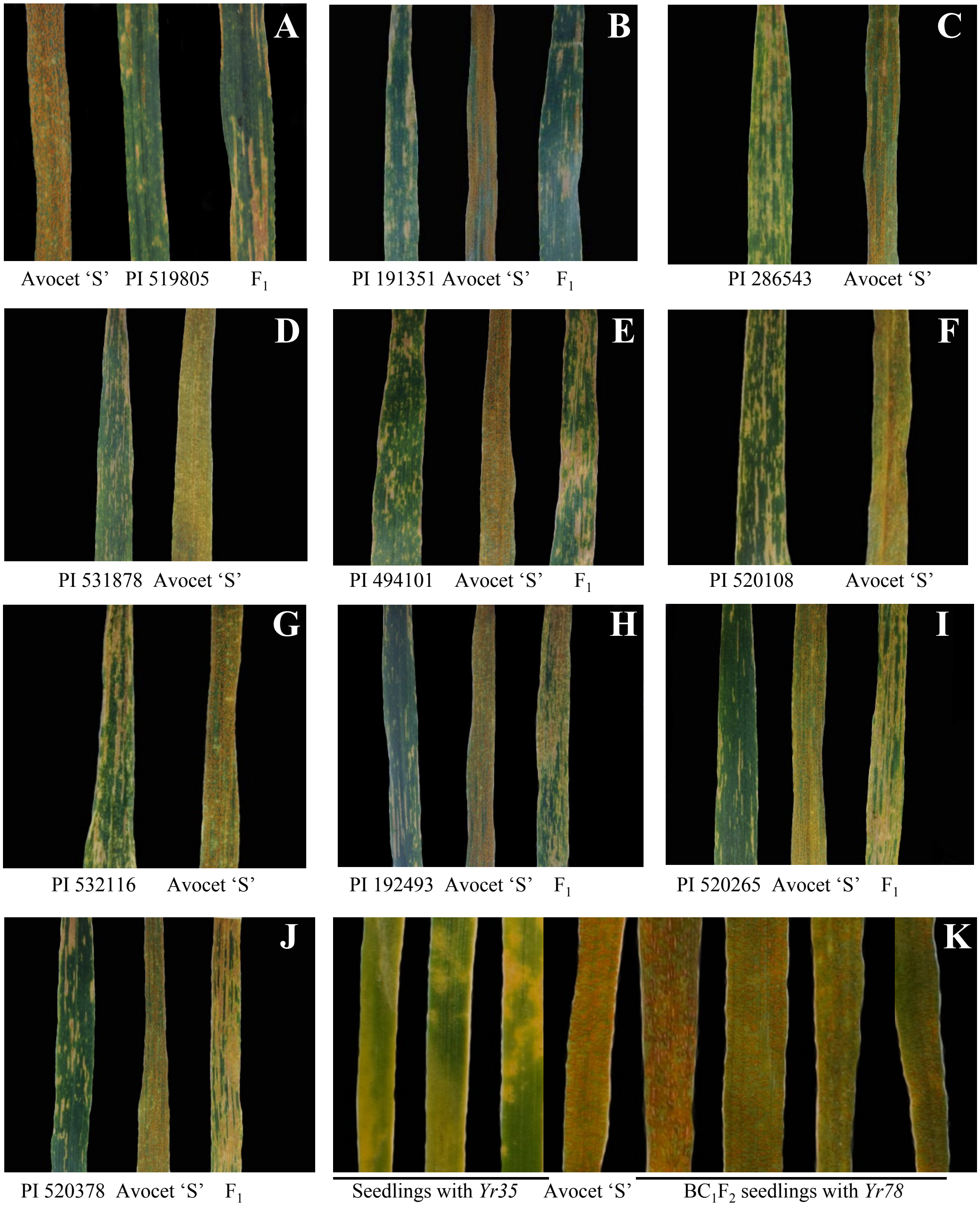

Supplementary Figure S1

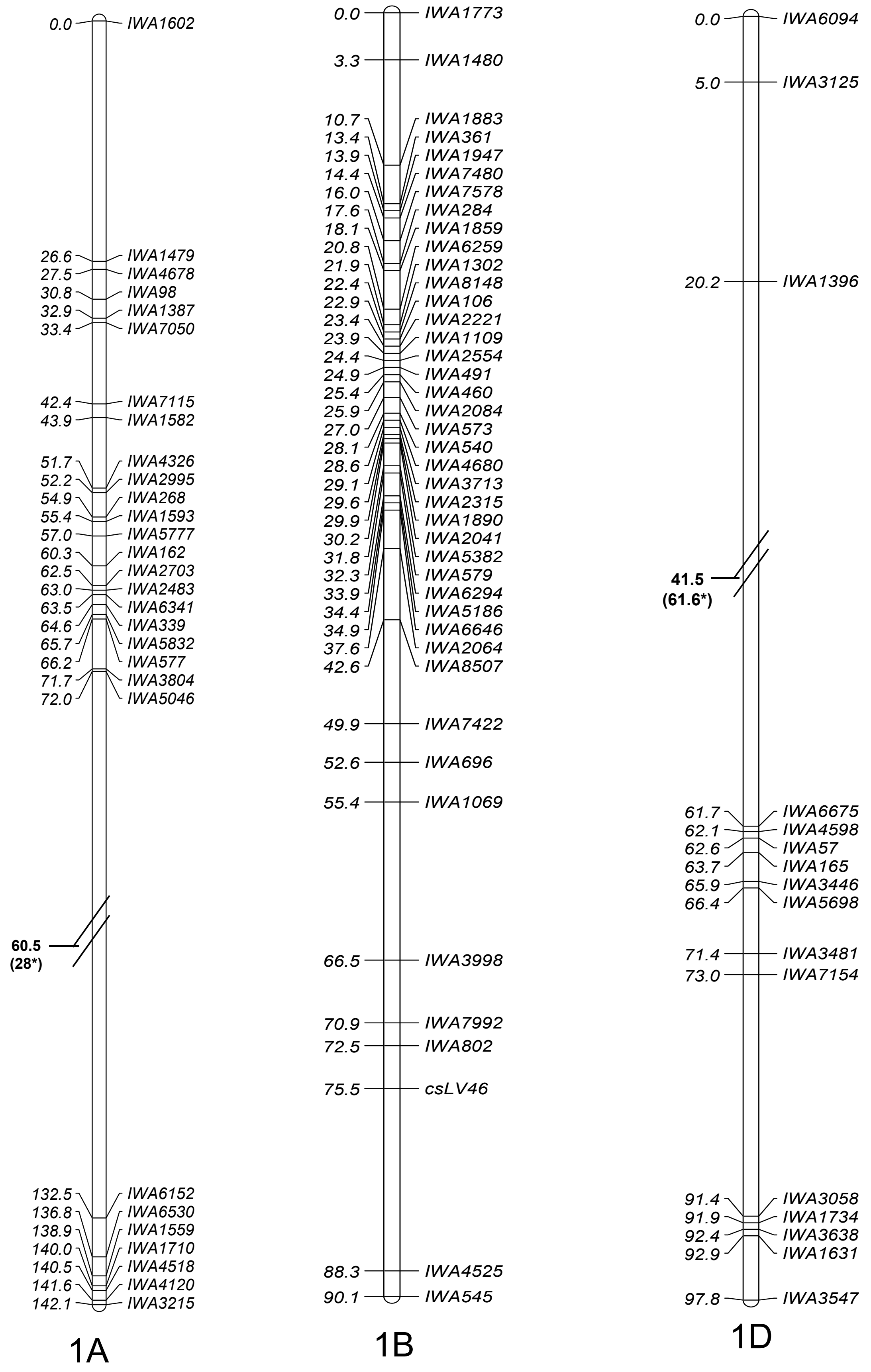

Supplementary Figure S2

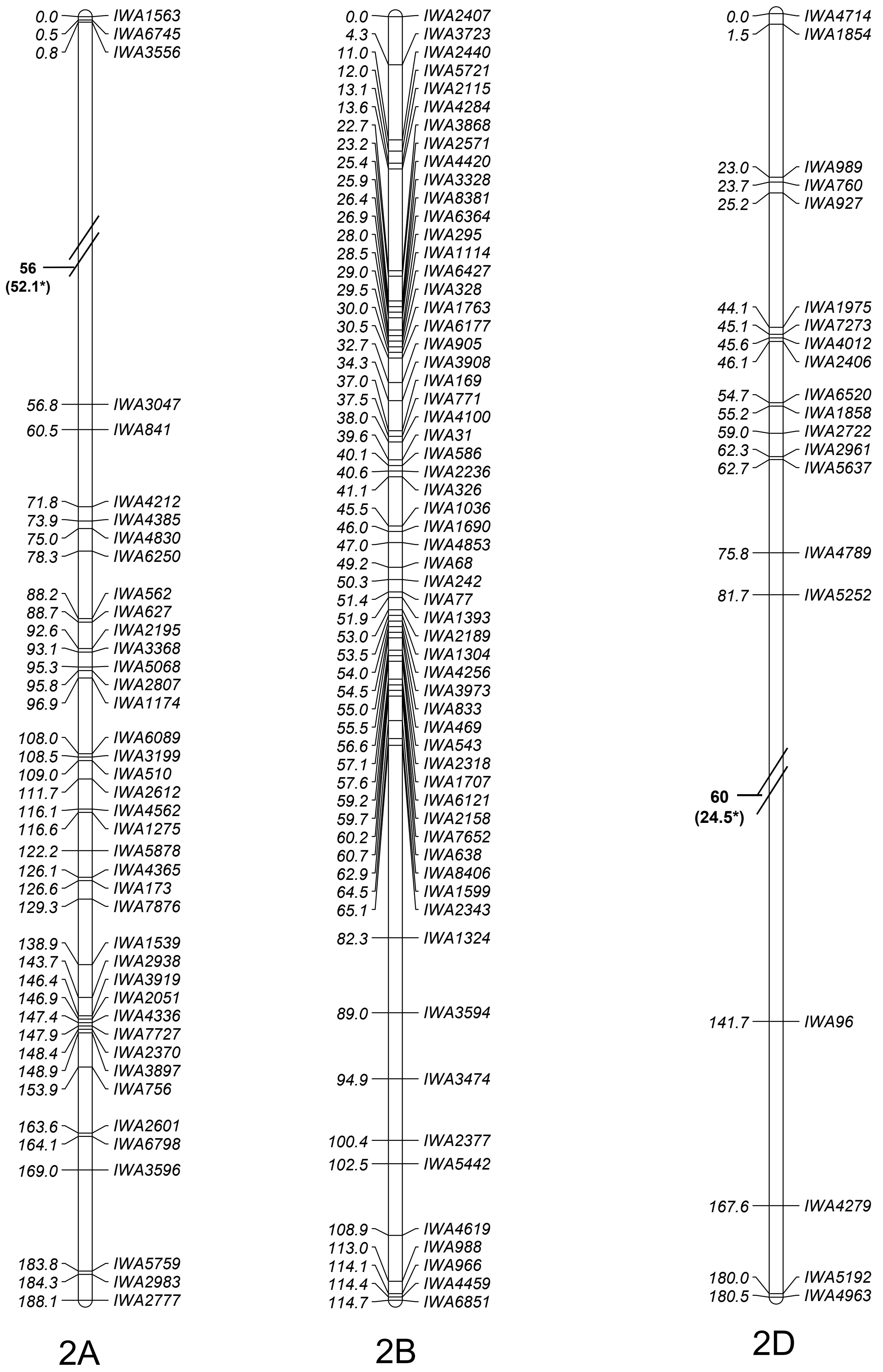

Supplementary Figure S2

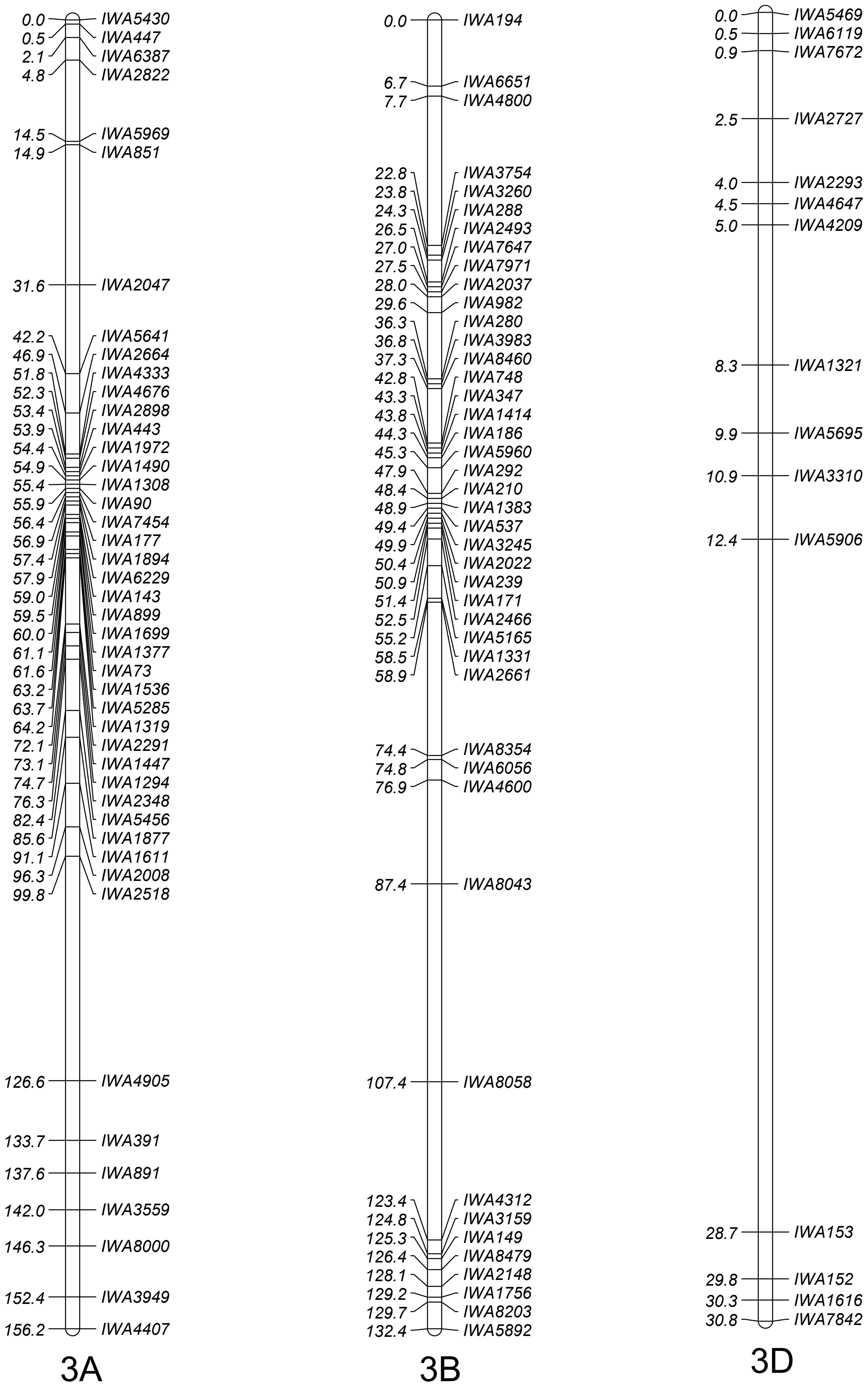

Supplementary Figure S2

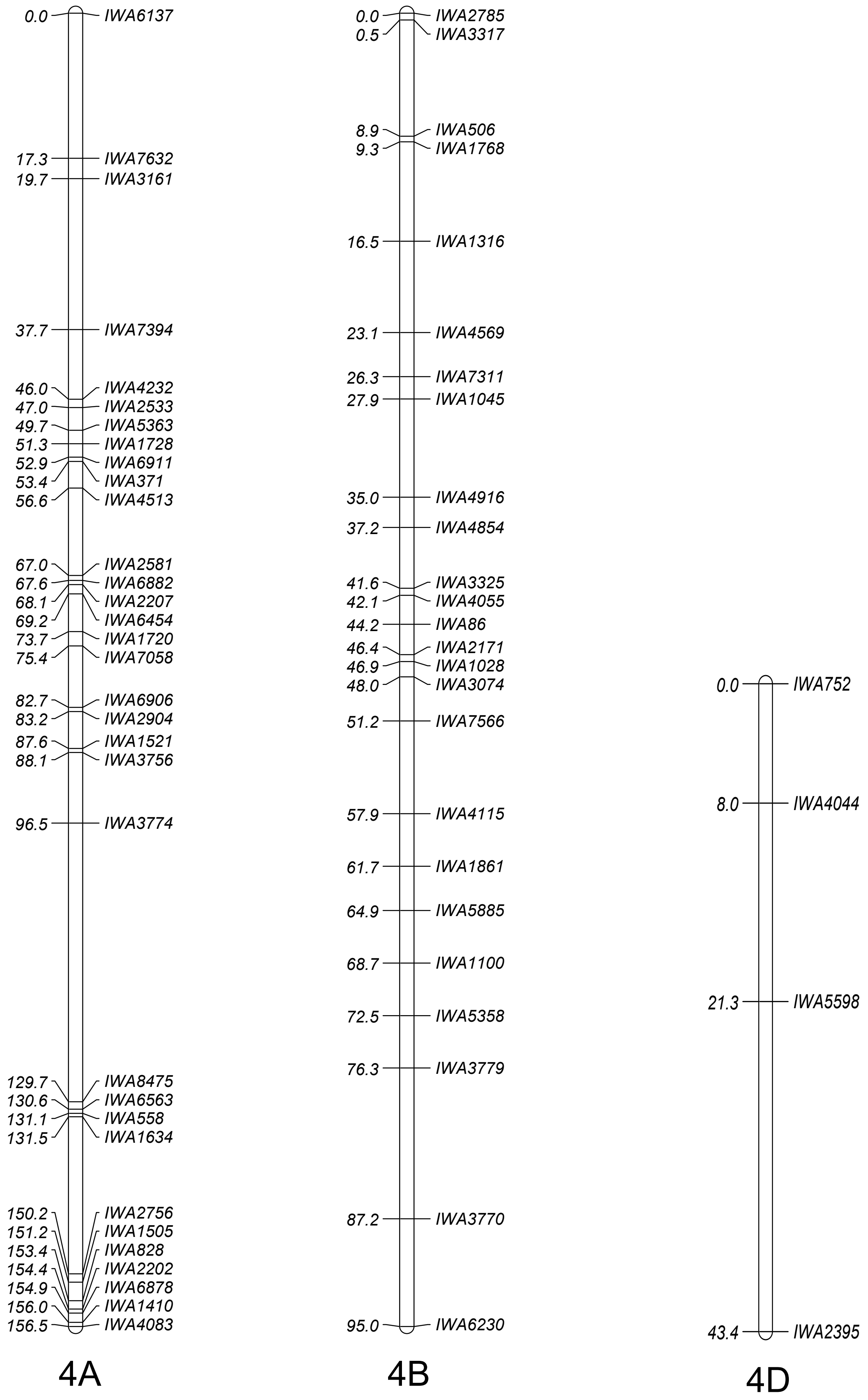

Supplementary Figure S2

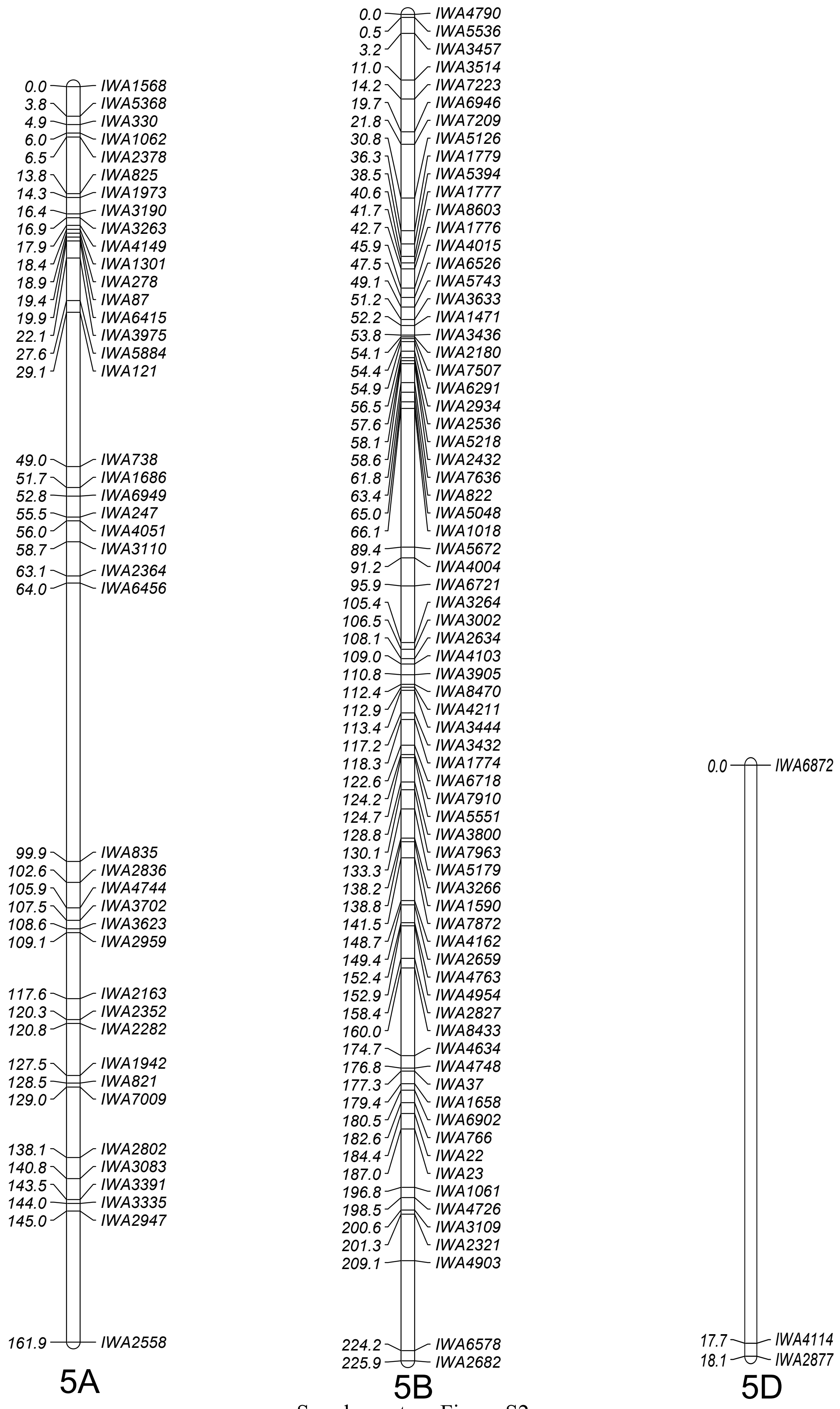

Supplementary Figure S2

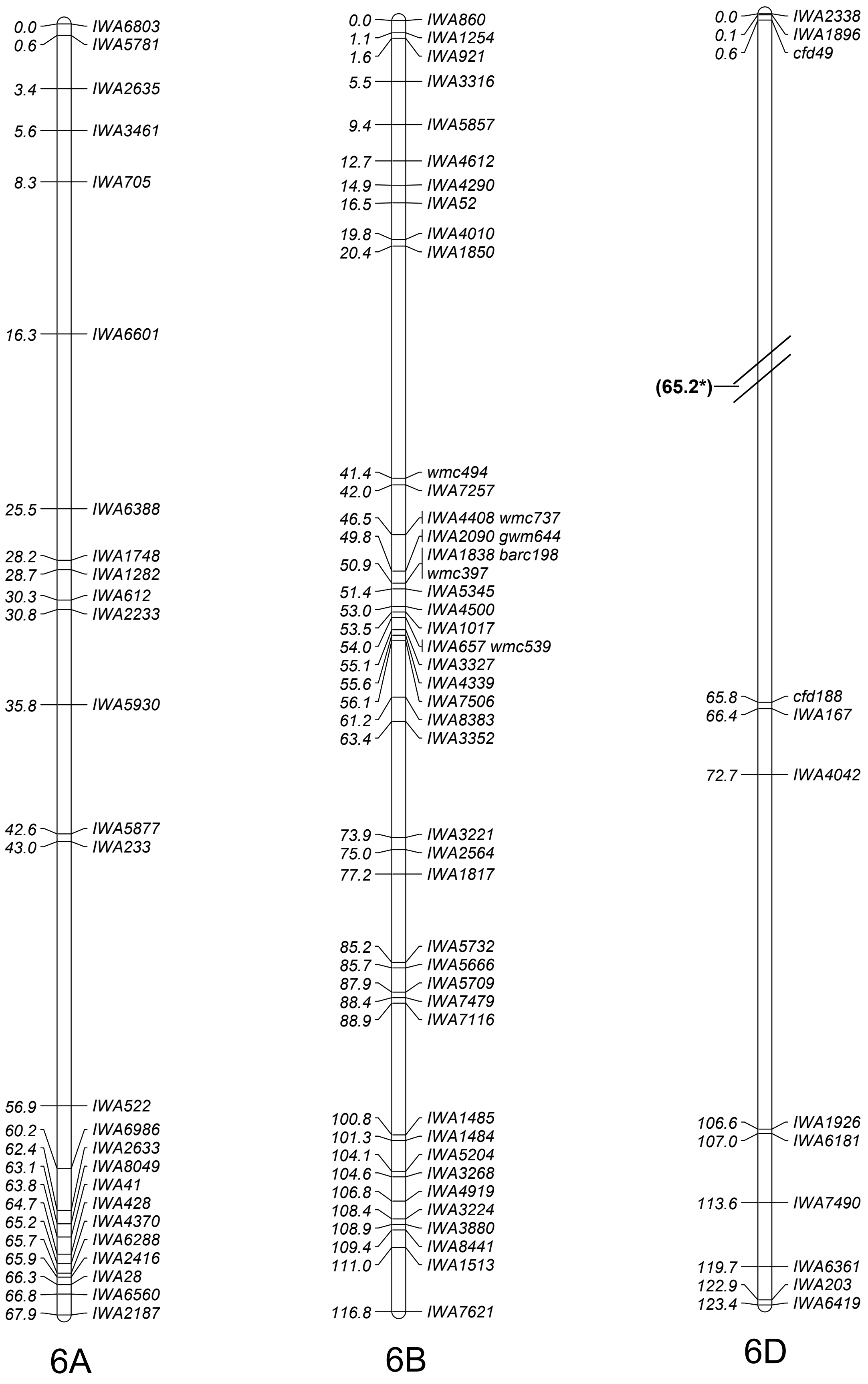

Supplementary Figure S2

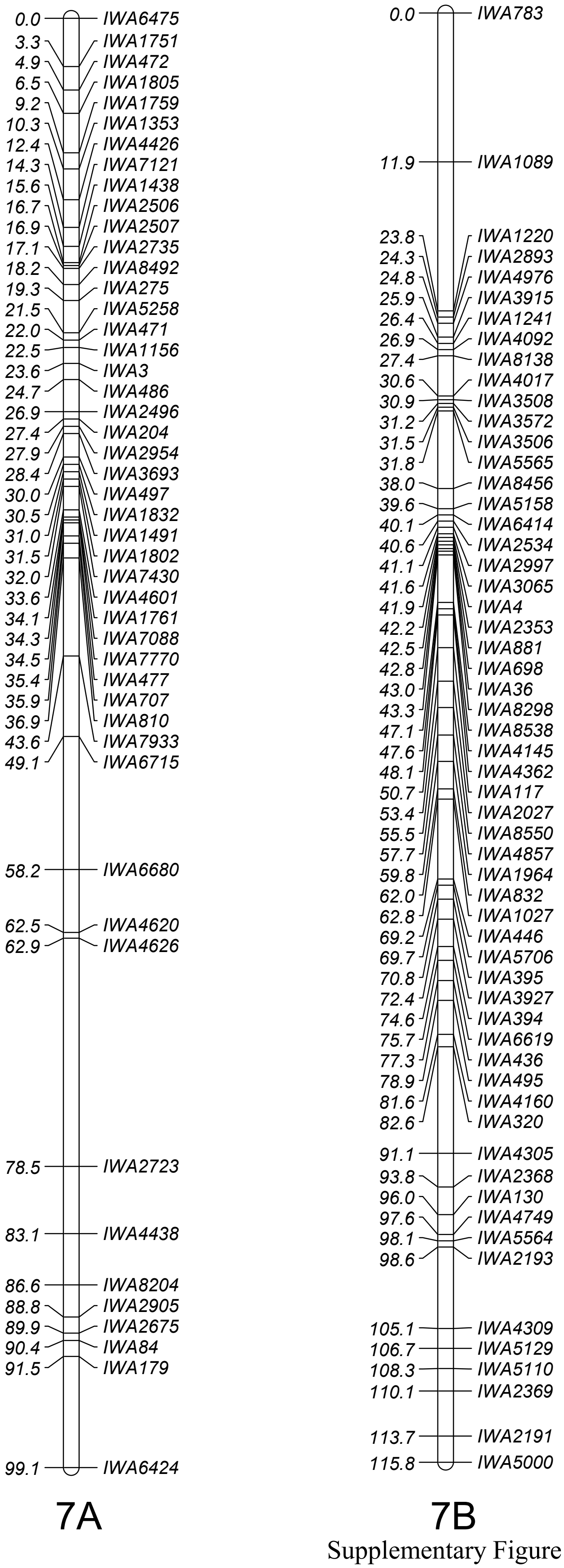

Supplementary Figure S2

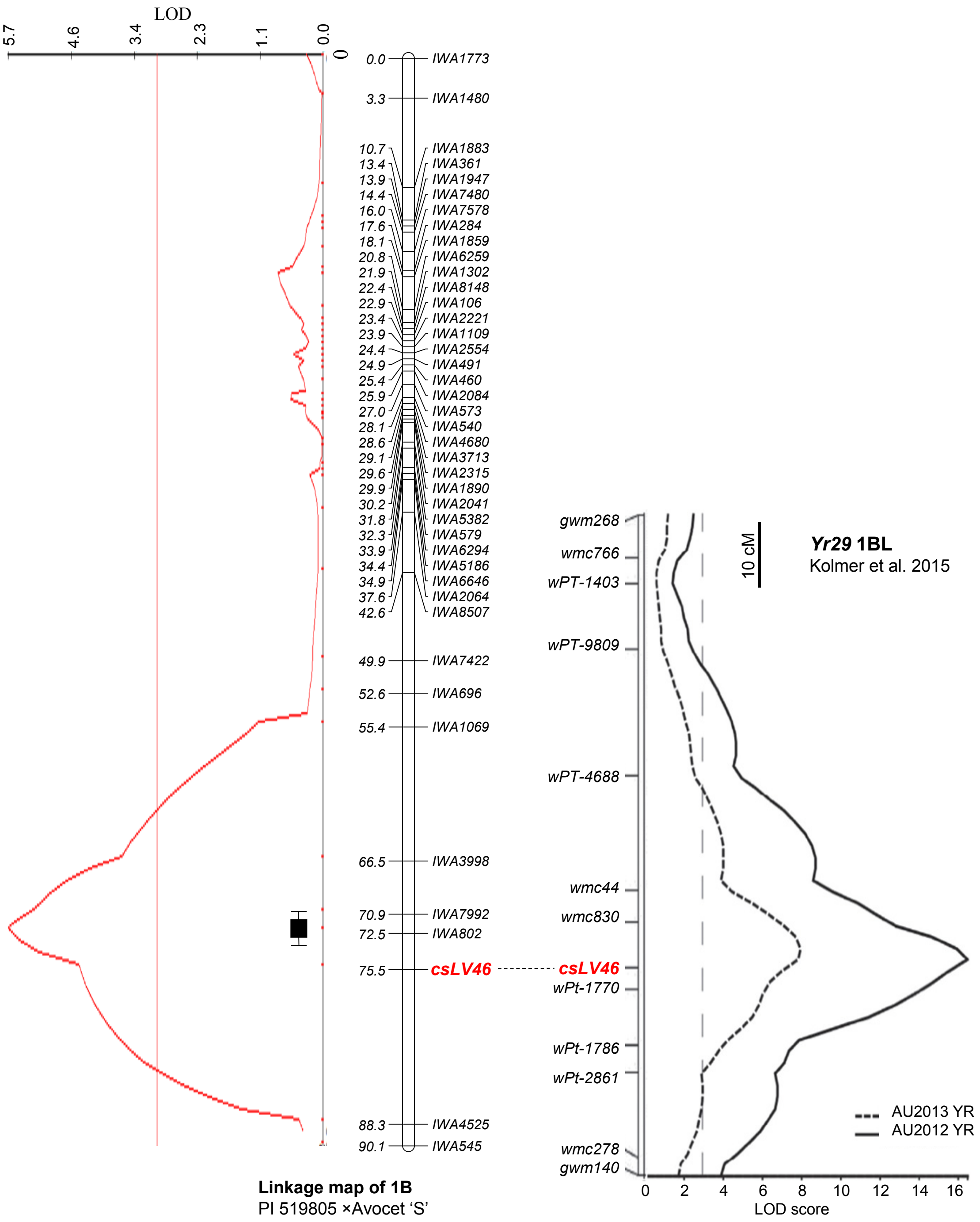

Supplementary Figure S3
